# The relation of synaptic biomarkers with Aβ, tau, glial activation, and neurodegeneration in Alzheimer’s disease

**DOI:** 10.1186/s40035-024-00420-1

**Published:** 2024-05-28

**Authors:** Yi-Ting Wang, Nicholas J. Ashton, Stijn Servaes, Johanna Nilsson, Marcel S. Woo, Tharick A. Pascoal, Cécile Tissot, Nesrine Rahmouni, Joseph Therriault, Firoza Lussier, Mira Chamoun, Serge Gauthier, Ann Brinkmalm, Henrik Zetterberg, Kaj Blennow, Pedro Rosa-Neto, Andréa L. Benedet

**Affiliations:** 1 Translational Neuroimaging Laboratory, McGill Centre for Studies in Aging, McGill University, Montreal, QC Canada; 2 Department of Psychiatry and Neurochemistry, Institute of Neuroscience & Physiology, the Sahlgrenska Academy at the University of Gothenburg, Mölndal, Sweden; 3 Centre for Age-Related Medicine, Stavanger University Hospital, Stavanger, Norway; 4 King’s College London, Institute of Psychiatry, Psychology and Neuroscience, Maurice Wohl Institute Clinical Neuroscience Institute, London, UK; 5 NIHR Biomedical Research Centre for Mental Health and Biomedical Research Unit for Dementia at South London and Maudsley NHS Foundation, London, UK; 6 Department of Neurology, University Medical Center Hamburg-Eppendorf, Hamburg, Germany; 7 Institute of Neuroimmunology and Multiple Sclerosis (INIMS), University Medical Center Hamburg-Eppendorf, Hamburg, Germany; 8 Department of Psychiatry, University of Pittsburgh, Pittsburgh, PA USA; 9 Department of Neurology, University of Pittsburgh, Pittsburgh, PA USA; 10 Alzheimer’s Disease Research Unit, The McGill University Research Centre for Studies in Aging, Montreal, McGill University, Montreal, QC Canada; 11 Clinical Neurochemistry Laboratory, Sahlgrenska University Hospital, Mölndal, Sweden; 12 Department of Neurodegenerative Disease, UCL Institute of Neurology, Queen Square, London, UK; 13 UK Dementia Research Institute at UCL, London, UK; 14 Hong Kong Center for Neurodegenerative Diseases, Clear Water Bay, Hong Kong, China; 15 Wisconsin Alzheimer’s Disease Research Center, University of Wisconsin School of Medicine and Public Health, University of Wisconsin-Madison, Madison, WI 53792 USA; 16 Montreal Neurological Institute, Montreal, QC Canada; 17 Department of Neurology and Neurosurgery, McGill University, Montreal, QC Canada

## Main text

Synaptic degeneration is a prominent feature of various neurodegenerative diseases and represents an early pathogenic event in Alzheimer’s disease (AD) [1, 2]. Multiple synapse-specific proteins involved in distinct synaptic pathways can be measured in the cerebrospinal fluid (CSF) and have been implicated as promising biomarkers of synaptic degeneration. Among them, the most extensively studied ones include the presynaptic proteins synaptosomal-associated protein-25 (SNAP25), growth-associated protein-43 (GAP43) and synaptotagmin-1 (SYT1) and postsynaptic protein neurogranin (NRGN) [3, 4]. However, the associations of these synaptic biomarkers with AD-related pathologies including amyloid-β (Aβ), tau, glial activity, neurodegeneration, and cognitive function are still not fully understood. The aim of this study was to investigate and compare the relationships of synaptic biomarkers with AD biomarker-informed pathophysiology and clinical disease progression.

This cross-sectional study included 144 participants from the Translational Biomarkers in Aging and Dementia (TRIAD) cohort, who had synaptic biomarkers quantified in the CSF and had undergone multimodal imaging assessments including structural magnetic resonance imaging (MRI), Aβ-positron emission tomography (PET) with [^18^F]AZD4694, and tau-PET with [^18^F]MK6240. In addition to the descriptive statistical analyses, linear regression models were performed to examine the association between biomarkers, correcting for age and sex in all analyses. Detailed descriptions of methods and statistical analysis are available in Additional file 1: Supplementary Material. Informed consent was obtained following ethics approval from the Institutional Review Board.

Clinical and demographic data are presented in Additional file 1: Table S1. Excluding the young participants, the average age of the population was 70 years old (± 7.74) and the AD dementia group was significantly younger than the other groups (*P* < 0.01). Concerning the synaptic biomarkers, age had a significant effect on SYT1 only (*P* = 0.004, partial eta-squared (*η*_*p*_^*2*^) = 0.06). We found no difference in the proportions of males and females between the groups (*P* = 0.39), although sex effect was observed on NRGN (*P* = 0.001, *η*_*p*_^*2*^ = 0.08 – when adjusting for covariates), where females had higher levels than males. Correlations between synaptic biomarkers and their concentrations across groups are presented in Tables S2 and S3, and Figures S1 and S2.

**Fig. 1:**
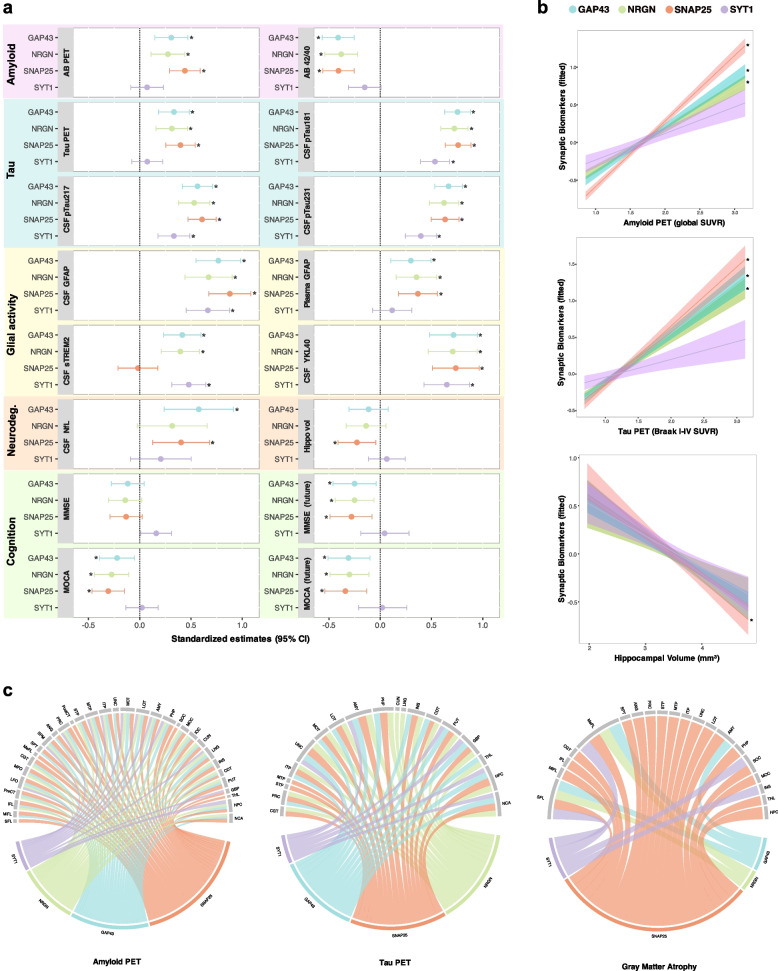
Associations of synaptic biomarkers with AD biomarkers and cognition. **a** Forest plot showing the standardized estimates of the linear regression models of associations between synaptic biomarkers and other CSF/imaging biomarkers, adjusted by age and sex (for future cognition, baseline scores were also accounted for in the models). Dots indicate β values and bars show the confidence intervals. **b** Associations between synaptic biomarkers and Aβ PET, Tau PET and hippocampal volume in participants within the AD continuum. The lines represent the linear regression, and the shaded areas show the 95% confidence interval. Symbol * indicates significant associations. **c** Chord diagrams showing the correlations between synaptic and imaging biomarkers in pre-defined anatomical brain regions that remained significant after FDR correction for multiple comparisons

When evaluating if Aβ pathology would predict synaptic biomarker levels, we found that high concentrations of synaptic biomarkers were significantly associated with increased Aβ pathology indexed by both Aβ PET and CSF Aβ42/40 (Fig. 1, Additional file 1: Figure S3 and Table S4) within the AD spectrum, except for SYT1, which did not associate with CSF Aβ42/40 or Aβ PET. From all biomarkers, both PET and CSF measures had the highest effect size on SNAP25, as indicated by the beta values of the regression models, although it was not significantly better than GAP43 or NRGN. These results were further reinforced by the voxel-wise analysis, with SNAP25 having more widespread associations with Aβ PET throughout the frontal, temporal, parietal and posterior cingulate cortices (Additional file 1: Fig. S4). Similarly, significant associations were also found between tau PET and synaptic biomarkers within the AD continuum, which were strongest for SNAP25 and weakest for SYT1. Stronger associations were found when CSF pTau181 was used as a proxy of tau pathology, with SNAP25 and GAP43 having the highest beta values. When tau PET uptake was evaluated at the voxel level, we observed its associations and correlations with GAP43, NRGN and SNAP25 are largely colocalizing on the same brain regions.

Next, we investigated the relationships between synaptic degeneration and glial activity, which was assessed with surrogate biomarkers of astrocytic reactivity and microglial activation, including glial fibrillary acidic protein (GFAP), chitinase-3-like protein 1 (YKL-40) and soluble triggering receptor expressed on myeloid cells 2 (sTREM2). We found that GFAP was positively associated with all synaptic biomarkers, and SNAP25 had the highest beta values in the regression models. Very similar findings were observed for YKL-40, but not with sTREM2, which emerged as a better predictor of SYT1 levels than other synaptic biomarker tested. Synaptic biomarkers also showed significant associations with neurodegeneration, indexed by CSF neurofilament light chain (NfL) and hippocampal volume. GAP43 showed the most substantial association with CSF NfL, as evidenced by its largest beta coefficient. However, hippocampal volume showed a significant inverse association only with SNAP25, which was also the biomarker that most correlated with grey matter atrophy in the pre-defined anatomical regions. Finally, we found that the synaptic biomarkers were significantly associated with cognition proxied by Montreal cognitive assessment (MOCA) but not by mini-mental state examination (MMSE) at the same time point. However, all biomarkers were able to predict future cognitive performance proxied by both MMSE and MOCA, except SYT1. Additional results of path analysis are presented in Table S5 and Figure S5.

The results of this study suggest that CSF synaptic biomarkers can indicate synaptic degeneration in cortical regions affected by Aβ and tau pathologies, and are also linked with glial activity and future cognitive deficit, reinforcing their potential use as a biomarker for synaptic dysfunction and degeneration in AD. Among the four synaptic biomarkers investigated, GAP43, NRGN and SNAP25 present very similar associations with Aβ and tau pathologies. SNAP25, in particular, has numerically greater associations and correlates with pathology in more brain regions. To our knowledge, no previous study had investigated the association of CSF synaptic biomarkers with the deposition of Aβ plaques and NFTs in the brain at the voxel level. In this study, voxel-based analyses demonstrated that synaptic biomarkers including GAP43, NRGN and SNAP25 showed positive associations with Aβ plaque deposition in multiple AD-related regions including temporal cortices, occipital cortices, precuneus, posterior cingulate, and medial orbitofrontal cortices. On the other hand, the association between the aforementioned synaptic biomarkers and NFT aggregation was primarily found in regions with early tau deposition including medial temporal and inferior-parietal cortices. Again, SNAP25 had numerically the strongest relationships with both Aβ-PET and tau-PET at the voxel level, although not significantly different from GAP43 and NRGN. Since studies have suggested that soluble forms of Aβ and tau impose direct toxicity on the synapses [5], it is important to investigate the associations of these synaptic biomarkers not only with plaques and tangles, but also with Aβ and p-tau in the CSF. Results from linear regression models suggested that higher concentrations of synaptic biomarkers were associated with a lower CSF Aβ42/40 ratio (indicating a higher concentration of Aβ deposition in the brain) and higher CSF p-tau concentrations. Among the CSF synaptic biomarkers investigated, SNAP25 presented the strongest effect size as indexed by beta coefficients, suggesting that SNAP25 rises at an early stage in the AD spectrum.

Beyond Aβ and tau pathologies, we also assessed whether synaptic biomarkers differ in their association with glial activity, neurodegeneration and cognition. In this study, GFAP was positively associated with all synaptic biomarkers although SNAP25 had numerically the highest association values. Very similar findings were obtained when YKL-40 was evaluated, but not with sTREM2. Interestingly, sTREM2 best predicted SYT1 levels, and no association was found with SNAP25. Preclinical studies provided evidence that microglia and astrocytes can drive synaptic degeneration in animal models of ageing and AD via ingestion of tagged synapses, contributing to cognitive decline [6–8]. Although knowledge of the involvement of astrocytes and microglia in synaptic ingestion in humans during ageing and AD remains very limited, results collectively suggest that glial cells are likely capable of inducing synaptic loss and degeneration in humans. Results from this study also showed significant associations of synaptic biomarkers with neurodegeneration biomarkers. A higher concentration of SNAP25 was significantly associated with smaller hippocampal volume and correlated with grey matter atrophy in several brain anatomical regions. In line with these findings, we also found synaptic biomarkers to predict future cognitive performance, proxied by MMSE and MOCA, whilst at baseline assessment, a significant association was only found between SNAP25 and MOCA.

These findings should be considered in light of some limitations such as the sample size and the inherent constraints of cross-sectional data analysis, which cannot implicate synaptic biomarker trajectories along disease progression. Furthermore, the availability of a PET ligand to study synaptic density [9, 10] would be very valuable to further investigate brain regional associations of synaptic degeneration and provide insights into the mechanistic links between AD biomarkers and synaptic alterations in the living human brains.

Overall, this study suggests that CSF synaptic biomarkers exhibit a degree of interchangeability in their potential utility. SNAP25 stands out as a superior CSF biomarker for assessing synaptic dysfunction due to its broader biomarker associations and larger effect sizes, supporting its potential inclusion in future AD clinical trials. This underscores the importance of considering synaptic function as a critical endpoint in AD treatment, beyond the traditional focus on Aβ and tau pathologies. Understanding the links between synaptic degeneration and other AD events offers insights for targeting synapses as a therapeutic opportunity in AD.

### Supplementary Information

**Additional file 1: Table S1.** Demographics and biomarker information. **Table S2.** Cross-correlation matrix between synaptic biomarkers (Spearman’s rho correlation coefficient). **Table S3.** Post hoc analysis (*Tukey* contrasts) of the biomarker concentrations across groups, accounting for age and sex. **Table S4.** Standardized estimates of the linear regression models of associations between synaptic biomarkers and other CSF/imaging biomarkers, adjusted by age and sex (for future cognition, baseline scores were also accounted for in the models). **Table S5.** Parameters and estimates of the path analysis. **Figure S1.** Biomarker concentration across groups – z-scores. **Figure S2.** Biomarker concentration across groups – raw values. **Figure S3.** Individual linear associations. **Figure S4.** Synaptic biomarkers and brain PET. **Figure S5.** Interconnection of biomarkers in the AD continuum.

## Data Availability

Data from the TRIAD cohort that support the findings of this study are available from the corresponding author upon reasonable request. All requests for raw and analyzed data and materials will be promptly reviewed by McGill University to verify if the request is subject to any intellectual property or confidentiality obligations. Anonymized data will be shared upon request from a qualified academic investigator for the purpose of replicating the procedures and results presented in this article. Any data and materials that can be shared will be released via a material transfer agreement. Data are not publicly available due to information that could compromise the privacy of research participants.

## Abbreviations

Aβ: Amyloid-beta
AD: Alzheimer's disease
CSF: Cerebrospinal fluid
GAP43: Growth-associated protein-43
GFAP: Glial fibrillary acidic protein
MMSE: Mini-mental state examination
MOCA: Montreal cognitive assessment
NfL: Neurofilament light chain protein
NRGN: Neurogranin
PET: Positron emission tomography
SNAP25: Synaptosomal-associated protein-25
SYT1: Synaptotagmin-1
TREM2: Triggering receptor expressed on myeloid cells 2
