# Supplementary material for: The relation of synaptic biomarkers with Aβ, tau, glial activation, and neurodegeneration in Alzheimer’s disease
Source: Transl Neurodegener. 2024 May 28;13:27. doi: 10.1186/s40035-024-00420-1 (PMC11131272; doi:10.1186/s40035-024-00420-1)
Supplement: Supplementary file 1 — Additional file 1: Table S1. Demographics and biomarker information. Table S2. Cross-correlation matrix between synaptic biomarkers (Spearman’s rho correlation coefficient). Table S3. Post hoc analysis (Tukey contrasts) of the biomarker concentrations across groups, accounting for age and sex. Table S4. Standardized estimates of the linear regression models of associations between synaptic biomarkers and other CSF/imaging biomarkers, adjusted by age and sex (for future cognition, baseline scores were also accounted for in the models). Table S5. Parameters and estimates of the path analysis. Figure S1. Biomarker concentration across groups – z-scores. Figure S2. Biomarker concentration across groups – raw values. Figure S3. Individual linear associations. Figure S4. Synaptic biomarkers and brain PET. Figure S5. Interconnection of biomarkers in the AD continuum. [file 40035_2024_420_MOESM1_ESM.docx]

**Supplementary Material**

**The relation of synaptic biomarkers with Aβ, tau, glial activation, and neurodegeneration in Alzheimer’s disease.**

Yi-Ting Wang^1^, Nicholas J. Ashton^2,3,4,5^, Stijn Servaes^1^, Johanna Nilsson^2^, Marcel S. Woo^1,6,7^, Tharick A. Pascoal^8,9^, Cécile Tissot^1^, Nesrine Rahmouni^1^, Joseph Therriault^1^, Firoza Lussier, Mira Chamoun^1^, Serge Gauthier^10^, Ann Brinkmalm^2^, Henrik Zetterberg^2,11,12,13,14,15^, Kaj Blennow^2,11^, Pedro Rosa-Neto^1,16,17^ and Andréa L. Benedet ^2^

**Contents**

| **Supplementary Methods** ……………………………………………………………………..  **Table S1.** Demographics and biomarker information …………………………………………  **Table S2.** Cross-correlation matrix between synaptic biomarkers ……………………………. | 2  5  6 |
| --- | --- |
| **Table S3.** Post hoc analysis of the biomarker concentrations across groups …………………. | 7 |
| **Table S4.** Results of the linear regression analysis ……………………………………………. | 8 |
| **Table S5.** Results of the path analysis ………………………………………………………… | 9 |
| **Figure S1.** Synaptic biomarker concentrations across groups – z-scored ……………………..  **Figure S2.** Synaptic biomarker concentrations across groups – raw values …………………..  **Figure S3.** Individual linear associations ……..……………………………………………….  **Figure S4.** Synaptic biomarkers and brain PET ……………………………………………….  **Figure S5.** Interconnection of biomarkers in the AD continuum.…………………………....... | 10  11  12  13  14 |

**Supplementary Methods**

**Study Design**

This cross-sectional study included participants from the Translational Biomarkers in Aging and Dementia (TRIAD) cohort. TRIAD is a biomarker-based study launched in 2017 as part of the McGill Centre for Studies in Aging. All participants underwent multimodal imaging assessments including structural magnetic resonance imaging (MRI), Aβ-PET with [^18^F]AZD4694, and tau-PET with [^18^F]MK6240. They also had biofluid collection and clinical and neuropsychological assessments, including the Mini-Mental State Examination and Clinical Dementia Rating (CDR). This study primarily includes individuals within the AD spectrum who have both synaptic biomarkers quantified in the CSF and PET imaging data available. For AD dementia participants, CDR scores were equal to or greater than 1, while those with mild cognitive impairment (MCI) had a CDR of 0.5, and cognitively unimpaired (CU) participants had a CDR of 0, all of whom met standard diagnostic criteria. For the initial descriptive analysis, the study sample consisted of 167 participants, including 26 young participants (<30 years old), 79 CU participants, 31 MCI participants, and 31 dementia participants, who were further categorized according to their brain Aβ status defined by visual rating of [^18^F]AZD4694 PET imaging. For subsequent analyses, participants with cognitive impairment who did not have evidence of Aβ pathology (non-AD) were excluded, leading to a sample size of 144 participants. The TRIAD study was approved by the Montreal Neurological Institute PET Working Committee and the Douglas Mental Health University Institute Research Ethics Board. Written informed consent was obtained from all participants.

**CSF Analysis**

CSF collection in the TRIAD cohort followed procedures previously described[^35^](https://sciwheel.com/work/citation?ids=9168410&pre=&suf=&sa=0&dbf=0). CSF data was analyzed at the University of Gothenburg, Sweden by scientists blinded to participant clinical information. CSF Aβ_40_, Aβ_42_, p-tau_181_ and total-tau concentrations were quantified with the LUMIPULSE G1200 (Fujirebio) assay, while p-tau_217_ and p-tau_231_ concentrations were measured using a custom Single molecule array (Simoa) assay (Quanterix, Billerica, MA, USA), as described previously[^36^](https://sciwheel.com/work/citation?ids=10115384&pre=&suf=&sa=0&dbf=0). For synaptic biomarkers, GAP43 and NRGN concentrations were measured using *in-house* immunoassays[^37,38^](https://sciwheel.com/work/citation?ids=965236,15289249&pre=&pre=&suf=&suf=&sa=0,0&dbf=0&dbf=0), whereas SNAP25 and SYT1 concentrations were quantified using an immunoprecipitation mass spectrometry method[^34^](https://sciwheel.com/work/citation?ids=14703916&pre=&suf=&sa=0&dbf=0). For glial activation/neuroinflammation biomarkers, CSF sTREM2 concentration was measured using an *in-house* electrochemiluminescence assay on the MesoScale Discovery SECTOR imager 6000 (MesoScale Discovery (MSD), Maryland, USA)[^18,39^](https://sciwheel.com/work/citation?ids=127192,1293225&pre=&pre=&suf=&suf=&sa=0,0&dbf=0&dbf=0). CSF YKL40 concentration was quantified with a commercial ELISA assay (R&D Systems, Minneapolis, MN) while GFAP concentration was quantified using a commercial single-plex assay (CSF) and a 4-plex assay (plasma) on the Simoa HD-X instrument (Quanterix, Billerica, MA, USA).

**Imaging Analysis**

All participants have acquired 3T T1-weighted MR images for structural analysis as well as co-registration purposes. T1-weighted images were segmented using the SPM12 tool, then non-linearly registered to the ADNI template using ANTS, as previously reported[^40^](https://sciwheel.com/work/citation?ids=14729661&pre=&suf=&sa=0&dbf=0). MRI images were also processed with an optimized Voxel-Based Morphometry (VBM) protocol. In addition to grey matter (GM) VBM, brain atrophy was estimated using hippocampal volume, which was adjusted for total intracranial volume (ICV). ICV adjustment was performed using data from cognitively unimpaired (CU) subjects at baseline.

PET imaging acquisition was performed with a Siemens High Resolution Research Tomograph. For Aβ PET, [^18^F]AZD4694 images were acquired at 40–70 min after the intravenous bolus injection of the tracer and reconstructed with an ordered subset expectation maximization (OSEM) algorithm on a four-dimensional (4D) volume with 3 frames (3 × 600 s). For tau PET, [^18^F]MK-6240 images were acquired at 90–110 min after the intravenous bolus injection of the tracer and reconstructed using the same OSEM algorithm on a 4D volume with 4 frames (4 × 300 s). At the end of each PET emission acquisition, a 6-min transmission scan with a rotating ^137^Cs point source was performed for attenuation correction. PET images were also corrected for motion, dead time, decay and scattered and random coincidences. Briefly, PET images were linearly registered to the native T1-weighted MRI and MRIs were linearly and nonlinearly registered to the ADNI standardized space. Then, PET images in the T1 space were brought to the ADNI standardized space using transformations from native MRI to the ADNI standardized space. PET images were subsequently spatially smoothed to an 8-mm full-width at half maximum resolution (FWHM)[^41^](https://sciwheel.com/work/citation?ids=9977914&pre=&suf=&sa=0&dbf=0). The whole cerebellum and inferior cerebellum gray matter were used as the reference regions for [^18^F]AZD4694 and [^18^F]MK6240, respectively. Tau PET SUVR was estimated from a composite area including the transentorhinal (Braak I-II) and limbic (Braak III-IV) cortices[^42^](https://sciwheel.com/work/citation?ids=13194202&pre=&suf=&sa=0&dbf=0). Neocortical [^18^F]AZD4694 SUVR value was estimated for each participant by averaging the SUVR from the precuneus, prefrontal, orbitofrontal, parietal, temporal, anterior and posterior cingulate cortices[^43^](https://sciwheel.com/work/citation?ids=9792713&pre=&suf=&sa=0&dbf=0). For PET and GM VBM, additional average quantifications were done for 68 brain anatomical regions as segmented in the ANIMAL atlas[^44^](https://sciwheel.com/work/citation?ids=15225969&pre=&suf=&sa=0&dbf=0). Within each of these regions, the top and bottom 5% voxels with extreme volumes were trimmed out before averaging left and right sides, resulting in 34 regions assessed (Supplementary material).

**Statistical Analysis**

All non-imaging statistical analyses were performed using the R software (version 4.0). Cross-sectional demographic and clinical data were assessed with *t* tests and *χ^2^* tests. Spearman rank correlation tests were applied to evaluate the association between biomarkers. Fluid biomarkers were log-transformed when needed. Linear regression models also tested the association between biomarkers, always adjusting by age and sex. Similar models were also applied to evaluate group differences and, when necessary, the Tukey honestly significant difference (HSD) test was used in the post hoc analysis.

Voxel-wise analyses were performed using RMINC, a R toolkit for statistical analysis of neuroimaging data. Initially, voxel-based linear regression models evaluated the associations between synaptic biomarkers and imaging outcomes, adjusting for age and sex. Random field theory (RFT) was used to correct the resulting *t-*parametric maps for multiple comparisons. For the subsequent analysis with PET, an ROI-based average SUVR was obtained by using a mask encompassing the brain voxels that were significantly associated with 3 out of the 4 synaptic biomarkers evaluated, as presented in the results. These ROI SUVRs were then used in linear regression models, as previously described, to obtain the standardized estimation values for each biomarker. Finally, Spearman was employed for each of the synaptic biomarkers to test their correlations with all 34 brain anatomical regions (for both PET tracers and GM VBM). The correlations that survived FDR correction were then plotted in a chord diagram (“*circlize*” package) where the density of correlations and their strength can be visually compared within and between biomarkers.

Given the significant associations and correlations between the majority of the biomarkers, we aimed to evaluate the complex associations between biomarkers altogether. We conducted a path analysis, which is a type of structural equation modelling, with full information maximum likelihood (FIML) estimation to test the fit of our hypothesized model using the R package ‘*lavaan*’, with bootstrapped standard errors calculated using 1000 draws. We then selected the “best” biomarker to represent each of the classes used in the model (Amyloid, Tau, Glial Activity, Synaptic, Neurodegeneration and Cognition). Following a biological framework, we hypothesized and tested the following model:

$$'Glial activity\sim Amyloid(CSF) + Age + Sex$$

$$Tau (CSF)\sim Amyloid(CSF) + Glial activity+ Age + Sex$$

$$Amyloid\left( PET \right)\sim Amyloid\left( CSF \right)+Tau(CSF)+Glial activity+ Age + Sex$$

$$Synaptic \sim Amyloid\left( CSF \right)+ Tau(CSF) + Glial activity + Age + Sex$$

$$Tau \left( PET \right)\sim Synaptic+ Tau(CSF) + Amyloid(PET) + Glial activity+ Age +Sex$$

$$Neurodegeneration \sim Tau (PET) + Synaptic + Glial activity + Age + Sex$$

$$Cognition \sim Tau (PET) + Neurodegeneration + Glial activity + Age + Sex'$$

The fit of our hypothesized model was assessed using the Root Mean Square Error of Approximation (RMSEA), Standardized Room Mean Square Residual (SRMR), Comparative Fit Index (CFI), and Tucker Lewis Index (TLI). We removed variables that were not significant in the paths resulting in a poor model fit. Values <0.05 and 0.08 indicate a good fit for the RMSEA and SRMR, respectively, and values >0.9 indicate a good fit for the CFI and TLI. Results are displayed in a diagram in which the distance and position of the nodes are arbitrarily defined.

**Table S1.** Demographics and biomarker information.

|  | **Young (*N*=26)** | **CU- (*N*=55)** | **CU+ (*N*=24)** | **MCI+ (*N*=19)** | **ADD  (*N*=20)** |
| --- | --- | --- | --- | --- | --- |
| **Sex (F)** | 16 (61.5) | 34 (61.8) | 15 (62.5) | 9 (47.4) | 8 (40.0) |
| **Age (years)** | 23.4 (1.91) | 71.1 (7.39) | 71.7 (7.66) | 71.80 (5.88) | 63.5 (7.44) |
| **NRGN^(a)^ (pg/mL)** | 187 (50.8) | 206 (114) | 252 (83.8) | 288 (106) | 258 (115) |
| **GAP43^(a)^ (pg/mL)** | 2490 (1260) | 3070 (1360) | 3240 (1460) | 3930 (1880) | 3780 (1650) |
| **SNAP25^(a)^ (pmol/L)** | 9.41 (8.42) | 12.80 (10.9) | 23.0 (11.6) | 29.3 (7.88) | 26.4 (21.7) |
| **SYT1^(a)^ (pmol/L)** | 37.8 (15.0) | 61.00 (23.4) | 65.8 (28.9) | 65.8 (29.7) | 58.5 (42.0) |
| **Amyloid PET (SUVR)** | 1.17 (0.08) | 1.26 (0.10) | 1.94 (0.43) | 2.39 (0.42) | 2.38 (0.45) |
| **Hippoc. Vol. (mm^3^)** | 4.11 (0.355) | 3.56 (0.35) | 3.55 (0.43) | 3.25 (0.36) | 2.84 (0.51) |
| **Tau PET (SUVR)** | 0.82 (0.08) | 0.90 (0.10) | 1.00 (0.15) | 1.56 (0.46) | 2.20 (0.61) |

Data are presented as count (%) or mean (SD), except for synaptic biomarkers (^a^) which were given in median (IQR).

*Abbreviations: ADD, Alzheimer’s disease dementia; CSF, cerebrospinal fluid; CU−, Aβ-negative cognitively unimpaired; CU+, Aβ-positive cognitively unimpaired; F, female; GAP43, growth-associated protein-43; Hippoc. Vol., hippocampal volume adjusted for intra-cranial volume; IQR, interquartile range; MCI+, Aβ-positive mild cognitive impairment; NRGN, Neurogranin; SD, Standard deviation; SNAP25, synaptosomal-associated protein-25; SYT1, synaptotagmin-1.*

**Table S2.** Cross-correlation matrix between synaptic biomarkers (Spearman’s rho correlation coefficient).

|  | **GAP43** | **NRGN** | **SNAP25** | **SYT1** |
| --- | --- | --- | --- | --- |
| **GAP43** | 1 |  |  |  |
| **NRGN** | 0.81^***^ | 1 |  |  |
| **SNAP25** | 0.652^***^ | 0.542^***^ | 1 |  |
| **SYT1** | 0.725^***^ | 0.6^***^ | 0.611^***^ | 1 |

^***^P<0.0001

*Abbreviations: GAP43, growth-associated protein-43; NRGN, Neurogranin; SNAP25, synaptosomal-associated protein-25; SYT1, synaptotagmin-1.*

**Table S3.** Post hoc analysis (*Tukey* contrasts) of the biomarker concentrations across groups, accounting for age and sex.

|  | **GAP43** | | **NRGN** | | **SNAP25** | | **SYT1** | |
| --- | --- | --- | --- | --- | --- | --- | --- | --- |
|  | *T* value | *P* value | *T* value | *P* value | *T* value | *P* value | *T* value | *P* value |
| CU- - Young | -0.431 | 0.992 | -0.319 | 0.997 | -0.170 | 1.000 | -1.089 | 0.792 |
| CU+ - Young | -0.392 | 0.994 | -0.129 | 1.000 | 0.846 | 0.905 | -0.932 | 0.870 |
| MCI+ - Young | 0.777 | 0.929 | 0.659 | 0.960 | 1.664 | 0.429 | -0.880 | 0.892 |
| ADD - Young | 0.738 | 0.940 | 1.106 | 0.783 | 1.604 | 0.467 | -1.278 | 0.679 |
| CU+ - CU- | 0.065 | 0.999 | 0.496 | 0.985 | 2.824 | 0.037 * | 0.325 | 0.997 |
| MCI+ - CU- | 3.065 | 0.018 * | 2.486 | 0.087 . | 4.732 | < 1e-04 *** | 0.400 | 0.994 |
| ADD - CU- | 2.568 | 0.072 . | 3.087 | 0.017 * | 3.809 | 0.001 ** | -0.163 | 1.000 |
| MCI+ - CU+ | 2.609 | 0.065 . | 1.763 | 0.370 | 1.863 | 0.315 | 0.089 | 1.000 |
| ADD - CU+ | 2.186 | 0.172 | 2.310 | 0.132 | 1.176 | 0.742 | -0.388 | 0.994 |
| ADD - MCI+ | -0.289 | 0.998 | 0.602 | 0.971 | -0.575 | 0.975 | -0.453 | 0.990 |

*Abbreviations: ADD, Alzheimer’s disease dementia; CU−, Aβ-negative cognitively unimpaired; CU+, Aβ-positive cognitively unimpaired; GAP43, growth-associated protein-43; MCI+, Aβ-positive mild cognitive impairment; NRGN, Neurogranin; SNAP25, synaptosomal-associated protein-25; SYT1, synaptotagmin-1.*

**Table S4.** Standardized estimates of the linear regression models of associations between synaptic biomarkers and other CSF/imaging biomarkers, adjusted by age and sex (for future cognition, baseline scores were also accounted for in the models).

| Class | Synaptic Biomarkers | Other Biomarkers | *β* Estimate | *P* value | CI 2.5 | CI 97.5 |
| --- | --- | --- | --- | --- | --- | --- |
| Amyloid | GAP43 | Aβ PET | 0.308 | **0.00023** | 0.147 | 0.469 |
| Amyloid | NRGN | Aβ PET | 0.275 | **0.00124** | 0.110 | 0.439 |
| Amyloid | SNAP25 | Aβ PET | 0.440 | **3.15E-08** | 0.292 | 0.589 |
| Amyloid | SYT1 | Aβ PET | 0.072 | 0.37241 | -0.087 | 0.231 |
| Amyloid | GAP43 | CSF Aβ42/40 | -0.410 | **6.60E-07** | -0.566 | -0.255 |
| Amyloid | NRGN | CSF Aβ42/40 | -0.380 | **6.23E-06** | -0.540 | -0.220 |
| Amyloid | SNAP25 | CSF Aβ42/40 | -0.407 | **5.11E-07** | -0.559 | -0.254 |
| Amyloid | SYT1 | CSF Aβ42/40 | -0.151 | 0.06134 | -0.309 | 0.007 |
| Tau | GAP43 | CSF pTau181 | 0.753 | **6.36E-23** | 0.628 | 0.879 |
| Tau | NRGN | CSF pTau181 | 0.723 | **5.77E-20** | 0.590 | 0.856 |
| Tau | SNAP25 | CSF pTau181 | 0.760 | **5.59E-23** | 0.634 | 0.886 |
| Tau | SYT1 | CSF pTau181 | 0.534 | **8.90E-12** | 0.392 | 0.676 |
| Tau | GAP43 | CSF pTau217 | 0.563 | **3.94E-12** | 0.417 | 0.710 |
| Tau | NRGN | CSF pTau217 | 0.531 | **1.85E-10** | 0.378 | 0.683 |
| Tau | SNAP25 | CSF pTau217 | 0.608 | **5.35E-15** | 0.471 | 0.745 |
| Tau | SYT1 | CSF pTau217 | 0.333 | **3.87E-05** | 0.178 | 0.487 |
| Tau | GAP43 | CSF pTau231 | 0.667 | **1.56E-17** | 0.532 | 0.801 |
| Tau | NRGN | CSF pTau231 | 0.623 | **1.39E-14** | 0.480 | 0.766 |
| Tau | SNAP25 | CSF pTau231 | 0.633 | **5.48E-16** | 0.497 | 0.769 |
| Tau | SYT1 | CSF pTau231 | 0.396 | **7.20E-07** | 0.245 | 0.547 |
| Tau | GAP43 | Tau PET | 0.334 | **2.29E-05** | 0.183 | 0.485 |
| Tau | NRGN | Tau PET | 0.313 | **9.53E-05** | 0.159 | 0.467 |
| Tau | SNAP25 | Tau PET | 0.397 | **1.92E-07** | 0.254 | 0.540 |
| Tau | SYT1 | Tau PET | 0.073 | 0.33928 | -0.078 | 0.224 |
| Glial activation | GAP43 | CSF GFAP | 0.766 | **1.25E-10** | 0.549 | 0.984 |
| Glial activation | NRGN | CSF GFAP | 0.672 | **4.79E-08** | 0.442 | 0.902 |
| Glial activation | SNAP25 | CSF GFAP | 0.879 | **2.93E-14** | 0.674 | 1.083 |
| Glial activation | SYT1 | CSF GFAP | 0.664 | **5.27E-09** | 0.453 | 0.875 |
| Glial activation | GAP43 | CSF sTREM2 | 0.415 | **1.39E-05** | 0.233 | 0.597 |
| Glial activation | NRGN | CSF sTREM2 | 0.396 | **4.47E-05** | 0.210 | 0.582 |
| Glial activation | SNAP25 | CSF sTREM2 | -0.017 | 0.85969 | -0.211 | 0.176 |
| Glial activation | SYT1 | CSF sTREM2 | 0.478 | **6.29E-08** | 0.313 | 0.643 |
| Glial activation | GAP43 | CSF YKL40 | 0.715 | **1.36E-08** | 0.481 | 0.948 |
| Glial activation | NRGN | CSF YKL40 | 0.707 | **3.17E-08** | 0.469 | 0.946 |
| Glial activation | SNAP25 | CSF YKL40 | 0.737 | **2.73E-09** | 0.508 | 0.966 |
| Glial activation | SYT1 | CSF YKL40 | 0.650 | **5.03E-08** | 0.427 | 0.872 |
| Glial activation | GAP43 | plasma GFAP | 0.299 | **0.00297** | 0.104 | 0.495 |
| Glial activation | NRGN | plasma GFAP | 0.354 | **0.00054** | 0.157 | 0.552 |
| Glial activation | SNAP25 | plasma GFAP | 0.367 | **0.00021** | 0.177 | 0.558 |
| Glial activation | SYT1 | plasma GFAP | 0.117 | **0.230877** | -0.075 | 0.308 |
| Neurodegeneration | GAP43 | CSF NfL | 0.576 | **0.00105** | 0.238 | 0.914 |
| Neurodegeneration | NRGN | CSF NfL | 0.316 | 0.06948 | -0.026 | 0.658 |
| Neurodegeneration | SNAP25 | CSF NfL | 0.403 | **0.00501** | 0.125 | 0.681 |
| Neurodegeneration | SYT1 | CSF NfL | 0.205 | 0.173632 | -0.092 | 0.502 |
| Neurodegeneration | GAP43 | Hippo vol | -0.113 | 0.24402 | -0.305 | 0.078 |
| Neurodegeneration | NRGN | Hippo vol | -0.138 | 0.16170 | -0.331 | 0.056 |
| Neurodegeneration | SNAP25 | Hippo vol | -0.227 | **0**.**01643** | -0.411 | -0.042 |
| Neurodegeneration | SYT1 | Hippo vol | 0.064 | 0.480456 | -0.115 | 0.243 |
| Cognition | GAP43 | MMSE | -0.117 | 0.15285 | -0.277 | 0.044 |
| Cognition | NRGN | MMSE | -0.142 | 0.08607 | -0.303 | 0.020 |
| Cognition | SNAP25 | MMSE | -0.133 | 0.09693 | -0.290 | 0.024 |
| Cognition | SYT1 | MMSE | 0.161 | **0.035462** | 0.011 | 0.311 |
| Cognition | GAP43 | MMSE (future) | -0.207 | **0.02457** | -0.387 | -0.027 |
| Cognition | NRGN | MMSE (future) | -0.239 | **0.01561** | -0.432 | -0.046 |
| Cognition | SNAP25 | MMSE (future) | -0.248 | **0.00643** | -0.424 | -0.071 |
| Cognition | SYT1 | MMSE (future) | 0.043 | 0.607703 | -0.122 | 0.207 |
| Cognition | GAP43 | MOCA | -0.221 | **0.01109** | -0.391 | -0.051 |
| Cognition | NRGN | MOCA | -0.274 | **0.00151** | -0.441 | -0.107 |
| Cognition | SNAP25 | MOCA | -0.307 | **0.00019** | -0.465 | -0.149 |
| Cognition | SYT1 | MOCA | 0.022 | 0.786960 | -0.137 | 0.180 |
| Cognition | GAP43 | MOCA (future) | -0.255 | **0.00552** | -0.433 | -0.077 |
| Cognition | NRGN | MOCA (future) | -0.291 | **0.00316** | -0.482 | -0.100 |
| Cognition | SNAP25 | MOCA (future) | -0.293 | **0.00120** | -0.468 | -0.119 |
| Cognition | SYT1 | MOCA (future) | 0.032 | 0.699659 | -0.133 | 0.197 |
|  |  |  |  |  |  |  |

Abbreviations: Aβ PET, Amyloid positron emission tomography; CI, confidence interval; CSF, cerebrospinal fluid; GAP43, growth-associated protein-43; GFAP, glial fibrillary acidic protein; Hippo. Vol, hippocampal volume adjusted for intra-cranial volume; MMSE, mini-mental state examination; MOCA, Montreal cognitive assessment; NRGN, Neurogranin; NfL, neurofilament light chain protein; pTau, phosphorylated Tau; SNAP25, synaptosomal-associated protein-25; sTREM2, soluble Triggering receptor expressed on myeloid cells 2; SYT1, synaptotagmin-1; YKL40, Chitinase-3-like protein 1.

**Table S5.** Parameters and estimates of the path analysis.

|  | **Full Model** | | **Reduced Model** | |
| --- | --- | --- | --- | --- |
|  | ***β* Estimate (Std.Err.)** | ***P* value** | ***β* Estimate (Std.Err.)** | ***P* value** |
|  |  |  |  |  |
| **Regression Slopes** |  |  |  |  |
| *Glial* |  |  |  |  |
| Amy (CSF) | -0.23(0.05) | <0.0001 | -0.24(0.05) | <0.0001 |
| Age | 0.68(0.05) | <0.0001 | 0.69(0.05) | <0.0001 |
| Sex | 0.09(0.10) | 0.361 |  |  |
| Tau (CSF) |  |  |  |  |
| Amy (CSF) | -0.56(0.05) | <0.0001 | -0.56(0.05) | <0.0001 |
| Glial | 0.57(0.07) | <0.0001 | 0.57(0.07) | <0.0001 |
| Age | -0.19(0.07) | 0.005 | -0.19(0.07) | 0.006 |
| Sex | -0.08(0.09) | 0.357 |  |  |
| *Amy (PET)* |  |  |  |  |
| Amy (CSF) | -0.56(0.07) | <0.0001 | -0.56(0.07) | <0.0001 |
| Tau (CSF) | 0.33(0.09) | <0.0001 | 0.32(0.07) | <0.0001 |
| Glial | -0.02(0.09) | 0.864 |  |  |
| Age | 0.01(0.08) | 0.930 |  |  |
| Sex | 0.00(0.09) | 0.987 |  |  |
| *Syn* |  |  |  |  |
| Amy (CSF) | 0.22(0.08) | 0.004 | 0.22(0.08) | 0.008 |
| Tau (CSF) | 0.80(0.10) | <0.0001 | 0.82(0.10) | <0.0001 |
| Glial | 0.33(0.11) | 0.002 | 0.21(0.07) | 0.004 |
| Age | -0.13(0.08) | 0.120 |  |  |
| Sex | 0.11(0.10) | 0.289 |  |  |
| *Tau (PET)* |  |  |  |  |
| Syn | -0.13(0.09) | 0.131 |  |  |
| Tau (CSF) | 0.62(0.12) | <0.0001 | 0.51(0.09) | <0.0001 |
| Amy (PET) | 0.33(0.08) | <0.0001 | 0.35(0.08) | <0.0001 |
| Glial | 0.01(0.11) | 0.923 |  |  |
| Age | -0.20(0.09) | 0.028 | -0.20(0.06) | 0.002 |
| Sex | 0.04(0.11) | 0.694 |  |  |
| *Ndg* |  |  |  |  |
| Tau (PET) | 0.14(0.07) | 0.043 | 0.21(0.06) | <0.0001 |
| Syn | 0.20(0.13) | 0.126 |  |  |
| Glial | 0.08(0.13) | 0.535 | 0.22(0.08) | 0.008 |
| Age | 0.57(0.09) | <0.0001 | 0.55(0.08) | <0.0001 |
| Sex | 0.17(0.11) | 0.111 | 0.22(0.10) | 0.028 |
| *Cog* |  |  |  |  |
| Tau (PET) | -0.61(0.07) | <0.0001 | -0.59(0.07) | <0.0001 |
| Ndg | -0.39(0.12) | 0.002 | -0.42(0.12) | 0.001 |
| Syn | 0.02(0.07) | 0.724 |  |  |
| Age | 0.30(0.11) | 0.005 | 0.33(0.11) | 0.002 |
| Sex | -0.19(0.12) | 0.103 |  |  |
| **Residual Variances** |  |  |  |  |
| Glial | 0.35(0.04) | <0.0001 | 0.35(0.04) | <0.0001 |
| Tau (CSF) | 0.26(0.03) | <0.0001 | 0.26(0.03) | <0.0001 |
| Amy (PET) | 0.31(0.04) | <0.0001 | 0.31(0.04) | <0.0001 |
| Syn | 0.37(0.04) | <0.0001 | 0.38(0.05) | <0.0001 |
| Tau (PET) | 0.43(0.05) | <0.0001 | 0.44(0.05) | <0.0001 |
| Ndg | 0.29(0.06) | <0.0001 | 0.25(0.03) | <0.0001 |
| Cog | 0.42(0.05) | <0.0001 | 0.42(0.05) | <0.0001 |
| Amy (CSF) | 0.99+ |  | 0.99+ |  |
| Age | 0.99+ |  | 0.99+ |  |
| Sex | 0.25+ |  | 0.25+ |  |
| **Residual Covariances** |  |  |  |  |
| Syn w/ Ndg | -0.15(0.06) | 0.025 |  |  |
| Amy (CSF) w/Age | -0.35+ |  | -0.35+ |  |
| Amy (CSF) w/Sex | -0.04+ |  | -0.04+ |  |
| Age w/Sex | 0.01+ |  | 0.01+ |  |
| Syn w/Cog |  |  | 0.01(0.04) | 0.739 |
| **Fit Indices** |  |  |  |  |
| χ2 | 10.14(8) | 0.26 | 26.56(21) | 0.19 |
| RMSEA | 0.04 |  | 0.04 |  |
| CFI | 1 |  | 0.99 |  |
| TLI | 0.99 |  | 0.99 |  |
| BIC | 1897.87 |  | 1849.68 |  |
| AIC | 1755.32 |  | 1745.73 |  |
| SRMR | 0.02 |  | 0.03 |  |
|  |  |  |  |  |

^+^Fixed parameter

For this analysis, Amy (CSF) was proxied by CSF Aβ42/40, Tau (CSF) was proxied by CSF pTau181, Glial was proxied by CSF GFAP, Syn was proxied by SNAP25, Ndg was proxied by CSF NfL and Cog was proxied by MOCA.

*Abbreviations: AIC, Aikaike Information Criterion; Amy, amyloid pathology; BIC, Bayesian Information Criterion; CFI, Comparative Fit Index; CSF, cerebrospinal fluid; Cog, cognition; Glial, glial activity; Ndg, neurodegeneration; PET, positron emission tomography; RMSEA, Root Mean Square Error of Approximation; SRMR, Standardized Room Mean Square Residual; Syn, synaptic; Tau, tau pathology; TLI, Tucker Lewis Index; χ2, Chi-square.*

**Figure S1.** Biomarker concentration across groups – z-scores.

Boxplots displaying levels of CSF synaptic biomarkers across groups. The boxplots depict the median (horizontal bar), 25th to 75th percentiles (hinges) and whiskers indicate 10th and 90th percentiles. Group comparisons (presented in the Supplementary Table S2) were computed with a one-way ANCOVA adjusting for age and sex. Tukey honestly significant difference (HSD) test was used for the post hoc pairwise comparisons. The * indicates, for each biomarker, the groups that are significantly different from CU group.

*Abbreviations: ADD, Alzheimer’s disease dementia; CU−, Aβ-negative cognitively unimpaired; CU+, Aβ-positive cognitively unimpaired; GAP43, growth-associated protein-43; MCI+, Aβ-positive mild cognitive impairment; NRGN, Neurogranin; SNAP25, synaptosomal-associated protein-25; SYT1, synaptotagmin-1.*

**Figure S2.** Biomarker concentration across groups – raw values.

Boxplots displaying levels of CSF synaptic biomarkers across groups. The boxplots depict the median (horizontal bar), 25th to 75th percentiles (hinges) and whiskers indicate 10th and 90th percentiles. Non-AD was included here for informative purposes only – these participants were not included in any of the statistical analyses.

*Abbreviations: ADD, Alzheimer’s disease dementia; CU−, Aβ-negative cognitively unimpaired; CU+, Aβ-positive cognitively unimpaired; GAP43, growth-associated protein-43; MCI+, Aβ-positive mild cognitive impairment; Non-AD, Aβ-negative cognitively impaired; NRGN, Neurogranin; SNAP25, synaptosomal-associated protein-25; SYT1, synaptotagmin-1.*

**

**Figure S3.** Individual linear associations.

Associations between synaptic biomarkers and Aβ PET, Tau PET and Hippocampal volume in participants within the AD continuum. The line represents the linear regression, and the shaded area shows the 95% confidence interval. Dots represent individual values before linear model fitting (unfitted, in grey) and after model fitting (fitted, in colours). Linear models had age and sex as covariates.

*Abbreviations: GAP43, growth-associated protein-43; NRGN, Neurogranin; PET, positron emission tomography; SNAP25, synaptosomal-associated protein-25; SUVR, standard uptake value ratio; SYT1, synaptotagmin-1.*

**

**Figure S4.** Synaptic biomarkers and brain PET.

The black panels on the left show the T-parametric maps on the significant voxel-wise associations between synaptic biomarkers and Amyloid (Aβ) PET (top) and tau PET (bottom). The final brain map (middle orange panels) shows the brain regions where at least three (orange) or four (yellow) of the synaptic biomarkers had significant overlapping associations with Aβ PET (top) and tau PET (bottom). On the right side, the forest plots show the effect of the association between synaptic biomarkers and the average Aβ PET (top) and tau PET (bottom) SUVR of the overlapping brain regions depicted in the middle orange panels. The * indicates significant associations.

**

**Figure S5. Interconnection of biomarkers in the AD continuum.**

To evaluate the relationship between biomarkers simultaneously and incorporate the potential effect of these biomarkers via multiple pathways, we have performed path analysis. Following a biological framework, we hypothesized and tested the model described above. After removing the variables that were not significant in each regression (see Table S5), the final model above showed a good fit as indicated by the different indices reported (RMSEA=0.04; SRMR=0.02; CFI=0.99, TLI=0.98). The final model indicates that Amyloid CSF (represented by Aβ42/40), Tau CSF (represented by pTau181) and Glial activation (proxied by CSF GFAP) independently affect synaptic biomarker levels (Synaptic; proxied by SNAP25).

The network plot shows significant associations between biomarkers which are linked by straight black arrows indicating the directions of the causal relationship. Curved double-headed arrows represent the unexplained or residual variances in the quantified variables that are not accounted for by the predictors in the model. Gray dotted linear arrows represent residual covariance. The values displayed are the standardized estimates and their significance is indicated by asterisks when calculated.

*Abbreviations: Amy, Aβ pathology; CSF, cerebrospinal fluid; Cog, cognition; Glial, glial activity; Ndg, neurodegeneration; PET, positron emission tomography; Syn, synaptic; Tau, tau pathology.*
